# Cell type-specific manifestations of cortical thickness heterogeneity in schizophrenia

**DOI:** 10.1038/s41380-022-01460-7

**Published:** 2022-02-10

**Authors:** Maria A. Di Biase, Michael P. Geaghan, William R. Reay, Jakob Seidlitz, Cynthia Shannon Weickert, Alice Pébay, Melissa J. Green, Yann Quidé, Joshua R. Atkins, Michael J. Coleman, Sylvain Bouix, Evdokiya E. Knyazhanskaya, Amanda E. Lyall, Ofer Pasternak, Marek Kubicki, Yogesh Rathi, Andrew Visco, Megan Gaunnac, Jinglei Lv, Raquelle I. Mesholam-Gately, Kathryn E. Lewandowski, Daphne J. Holt, Matcheri S. Keshavan, Christos Pantelis, Dost Öngür, Alan Breier, Murray J. Cairns, Martha E. Shenton, Andrew Zalesky

**Affiliations:** 1 Melbourne Neuropsychiatry Centre, Department of Psychiatry, The University of Melbourne and Melbourne Health, Carlton South, VIC Australia; 2 Department of Psychiatry, Brigham and Women’s Hospital, Harvard Medical School, Boston, MA USA; 3 School of Biomedical Sciences and Pharmacy, University of Newcastle, Newcastle, NSW Australia; 4 Centre for Brain and Mental Health Research, Hunter Medical Research Institute, Newcastle, NSW Australia; 5 Department of Child and Adolescent Psychiatry and Behavioral Science, Children’s Hospital of Philadelphia, Philadelphia, PA USA; 6 Department of Psychiatry, University of Pennsylvania, Philadelphia, PA USA; 7 Neuroscience Research Australia, Randwick, NSW Australia; 8 Discipline of Psychiatry and Mental Health, University of New South Wales, Sydney, NSW Australia; 9 Department of Neuroscience & Physiology, Upstate Medical University, Syracuse, NY USA; 10 Department of Anatomy and Physiology, School of Biomedical Sciences, The University of Melbourne, Melbourne, VIC Australia; 11 Department of Surgery, Royal Melbourne Hospital, Melbourne Medical School, The University of Melbourne, Melbourne, VIC Australia; 12 Department of Radiology, Brigham and Women’s Hospital, Harvard Medical School, Boston, MA USA; 13 Department of Psychiatry, Massachusetts General Hospital, Harvard Medical School, Boston, MA USA; 14 Department of Psychiatry, Indiana University School of Medicine, Indianapolis, IN USA; 15 School of Biomedical Engineering & Brain and Mind Centre, The University of Sydney, Camperdown, NSW Australia; 16 Harvard Medical School and Beth Israel Deaconess Medical Center, Boston, MA USA; 17 Division of Psychotic Disorders, McLean Hospital, Belmont, MA USA; 18 Department of Psychiatry, Harvard Medical School, Boston, MA USA; 19 Massachusetts General Hospital, Department of Psychiatry, Harvard Medical School, Boston, MA USA; 20 Athinoula A. Martinos Center for Biomedical Imaging, Charlestown, MA USA; 21 Melbourne School of Engineering, The University of Melbourne, Parkville, VIC Australia

**Keywords:** Neuroscience, Genetics, Molecular biology

## Abstract

Brain morphology differs markedly between individuals with schizophrenia, but the cellular and genetic basis of this heterogeneity is poorly understood. Here, we sought to determine whether cortical thickness (CTh) heterogeneity in schizophrenia relates to interregional variation in distinct neural cell types, as inferred from established gene expression data and person-specific genomic variation. This study comprised 1849 participants in total, including a discovery (140 cases and 1267 controls) and a validation cohort (335 cases and 185 controls). To characterize CTh heterogeneity, normative ranges were established for 34 cortical regions and the extent of deviation from these ranges was measured for each individual with schizophrenia. CTh deviations were explained by interregional gene expression levels of five out of seven neural cell types examined: (1) astrocytes; (2) endothelial cells; (3) oligodendrocyte progenitor cells (OPCs); (4) excitatory neurons; and (5) inhibitory neurons. Regional alignment between CTh alterations with cell type transcriptional maps distinguished broad patient subtypes, which were validated against genomic data drawn from the same individuals. In a predominantly *neuronal/endothelial subtype* (22% of patients), CTh deviations covaried with polygenic risk for schizophrenia (sczPRS) calculated specifically from genes marking neuronal and endothelial cells (*r* = −0.40, *p* = 0.010). Whereas, in a predominantly *glia/OPC subtype* (43% of patients), CTh deviations covaried with sczPRS calculated from glia and OPC-linked genes (*r* = −0.30, *p* = 0.028). This multi-scale analysis of genomic, transcriptomic, and brain phenotypic data may indicate that CTh heterogeneity in schizophrenia relates to inter-individual variation in cell-type specific functions. Decomposing heterogeneity in relation to cortical cell types enables prioritization of schizophrenia subsets for future disease modeling efforts.

## Introduction

Population averages of brain morphometric changes have dominated magnetic resonance imaging (MRI) studies in psychiatry. One of the most frequently reported MRI findings in schizophrenia is reduced cortical thickness (CTh) [1, 2]. However, recent studies report that CTh alterations do not converge to specific regional loci, most likely due to heterogeneity between individuals [3]. Interpreting this variability requires a strategy for dissecting the inherent complexity of MRI phenotypes [4], given that coarse measures of brain structure cannot adequately resolve distinct cellular processes.

One promising strategy is to systematically combine in vivo MRI measures with ex vivo gene expression data measured from postmortem brains [5–7]. Using this approach, transcriptomic correlates of case-control CTh differences in psychiatric disorders were recently identified [8]. Transcriptomic data can be annotated to specific neural cell classes, enabling the study of the cellular basis of large-scale morphometric pathology in schizophrenia [8]. However, inter-individual variation in cellular pathologies may manifest as marked CTh heterogeneity. Indeed, postmortem studies report considerable heterogeneity in the extent of neuronal and glial pathologies across schizophrenia-affected brains [9–11]. Therefore, using transcriptomics to elucidate the cellular basis of distinct profiles of CTh alterations can provide insight into person-specific sources of brain dysfunction in schizophrenia.

The present study builds on previous work revealing cellular correlates of group-level CTh alterations in schizophrenia [8]. Specifically, we extend inference to the individual level and ask whether CTh heterogeneity across individuals with schizophrenia relates to variation in interregional gene expression of specific neural cell types. To characterize CTh heterogeneity (inter-individual variation), normative ranges (i.e., centiles) in regional CTh [3, 12, 13] were defined on healthy controls as a function of age and sex, and CTh deviations were then estimated for each individual with schizophrenia. CTh deviations were systematically combined with regional gene expression maps of several neural cell-types. To investigate implicit biological links between individuals, each patient was classified into cell-based subtypes according to cell-patterned CTh deviations. Cell-based patient subtypes were then validated against genomic data drawn from the same individuals, with validation success operationalized as significant covariance between the severity of cell-linked CTh deviations and genetic liability ascribed to matched cell types. Biologically valid stratification of patients using cell-based approaches could usher in a new strategy to understand biological heterogeneity in schizophrenia and to address patient variability in planning in vitro disease models [5, 14].

## Methods

**Fig. 1:**
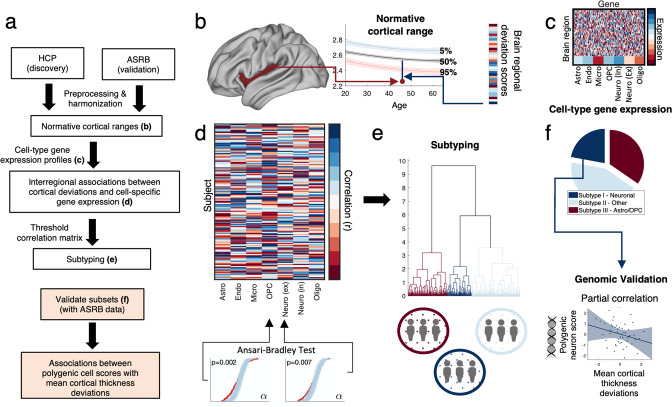
Schematic diagram of methodology. **a** Overview of the methodology and datasets. Note that steps b to d were completed for two independent datasets: Human Connectome Project (HCP; discovery) and Australian Schizophrenia Research Bank (ASRB; validation). **b** Quantile regression was used to establish normative ranges of individual CTh variation, given age and sex. Percentile curves (5%, 50%, 95%) are shown as a function of age for CTh in an example cortical region. Shading denotes 95% confidence intervals. For each cortical region, individuals with schizophrenia were compared to the normative range established for their age and sex. The vector shown provides a putative CTh deviation profile that summarizes individual differences across 34 brain regions. **c** Cell-class gene-expression brain maps reflected the mean expression of seven cell-type gene sets (columns) in each of the 34 (left hemisphere) cortical regions (rows). **d** For each patient (rows), a correlation coefficient (matrix elements) quantified the extent of spatial coupling between interregional cortical deviation scores and the 7 cell-class gene expression maps (columns). The Ansari-Bradley test was used to evaluate whether the observed distribution of correlations significantly differed from a null distribution. **e** Ward’s linkage was used to cluster individuals into broad cell-based subtypes based on spatial coupling between regional CTh deviations and regional transcriptomic maps. **f** Partial correlations evaluated whether polygenic cell scores (e.g., neurons) covaried with the severity of cortical thickness deviations among individuals comprising the broad cell-based subtypes.

This study was approved by the Melbourne Health Human Research Ethics Committee. All participants provided written informed consent for the analysis of their data. Figure 1 provides a schematic of the overall methodology.

### Datasets

This study comprised a total of 1849 participants drawn from three independent datasets: (1) the Human Connectome Project-Young Adult Sample [15]; (2) the Human Connectome Project for Early Psychosis (HCP-psychosis) [16]; and (3) the Australian Schizophrenia Research Bank (ASRB) [17]. The HCP-Young Adult Sample and HCP-psychosis datasets were acquired using very similar MRI protocols (see Supplementary Material), enabling them to be combined to form a single discovery cohort (*n* = 1267 comprising 140 cases [age: 22.78 ± 3.83; 48(34%) females] and 1127 controls [age: 28.50 ± 3.83); 598(53%) females]. The ASRB dataset was used to evaluate replicability and validity (*n* = 520 comprising 335 cases [age: 39.70 ± 10.81; 100(30%) females] and 185 controls [age: 41.05 ± 14.02); 94(51%) females]. Clinical and site characteristics for each dataset are shown in Supplementary Tables 1 and 2. Inclusion and exclusion criteria for each respective dataset are described elsewhere (HCP-Young adult [15]; HCP-Psychosis [16] and ASRB [17]).

### Deriving regional deviation profiles of CTh

MRI image acquisition, quality control procedures, CTh measurement and between-scan harmonization are described in Supplementary Material (Supplementary Fig. 1). Quantile regression [18] was used to obtain a normative range of regional CTh variation as a function of age and sex [3] (see Supplementary Material for model details). Individuals with schizophrenia were positioned on the normative percentile charts and then a continuous measure of deviation (Δ) from the established normative range was expressed as a z-score for each individual, reflecting the difference from mean CTh computed across all individuals in the training dataset. Quantile regression was repeated for each cortical region, resulting in a person-specific profile of regional CTh deviations (Fig. 1b).

### Mapping cell type-specific gene expression patterns in the human brain

There is currently no available means to measure cortical gene transcription in vivo. Furthermore, whole-brain gene expression data from schizophrenia-affected donors are not currently available. Therefore, we leverage anatomically resolved gene expression data (~500 tissue samples for ~15k genes) from six neurotypical postmortem brains (five males/one female, with ages ranging from 24 to 57 years) provided by the Allen Brain Institute [19] to approximate transcriptional landscapes of specific cortical regions and cell types. Alignment of these data to the left hemisphere of the Desikan-Killiany atlas is described elsewhere [20–22] and in the Supplementary Material. Genes were assigned to seven specific cell types using gene sets from single-cell studies of the adult human cortex (Fig. 1c) [23–27]. Cell types included (i) astrocytes; (ii) endothelial; (iii) microglial; (iv) oligodendrocyte progenitors; (v) excitatory neurons; (vi) inhibitory neurons; and (vii) oligodendrocytes. Supplementary Tables 3-5 and Supplementary Figure 2 report associations between schizophrenia and the seven gene sets. Mean expression of each cell type-specific gene set was determined in 34 Desikan–Killiany atlas regions (left hemisphere) and normalized (converted into z-scores) by mean expression across the entire brain. This resulted in seven cell type transcriptional maps, which estimated the regional distributions of gene expression for the seven cell types.

### Cell-type gene expression associations with CTh variation

We first sought to determine the extent of spatial coupling between *raw* CTh estimated from healthy controls and gene expression maps of each cell type. To this end, interregional levels of cell type-specific gene expression were correlated across the 34 cortical regions with mean CTh (computed across healthy controls). The false discovery rate (FDR) was used to enforce control over multiple comparisons (seven correlations corresponding to each cell type = 7 tests).

Next, the cellular correlates of CTh heterogeneity in schizophrenia were examined. Here, interregional levels of cell type-specific gene expression were correlated across the 34 cortical regions with individual *deviations* in regional CTh estimates (output from normative modeling; Fig. 1d). As the regional pattern of CTh deviations varied, we hypothesized that associations with specific cell types would only be evident in subsets of individuals. Therefore, the Ansari-Bradley test examined whether correlation distributions (across individuals) were more dispersed than a null distribution of correlation coefficients computed by randomizing the regional gene expression values (1000 permutations: Fig. 1d). On each permutation, regional gene expression values were randomized, thereby breaking any regional pattern in gene expression, and establishing a null condition. Rejection of the null hypothesis indicated that the spatial coupling between regional CTh deviations and a cell type transcriptional map was greater in *some* individuals than attributable to chance (FDR*p* < 0.05; i.e., dispersion in the inter-individual distribution of correlation coefficients was greater than expected due to chance). These analyses were conducted separately in the HCP (discovery) and ASRB (validation) cohorts to examine reproducibility.

### Defining cell-based patient subtypes from cell-patterned CTh deviations

For each dataset (i.e., Discovery and Validation), individuals with schizophrenia were classified into broad cell-based subtypes based on person-specific associations between their spatially patterned CTh deviation profile with interregional gene expression maps of specific cell types. To improve the clustering algorithm performance, dimensionality reduction with principal component analysis (PCA) was applied to the (subject x cell specific gene expression maps) correlation matrix, retaining a set of four principal components that captured the most variance, as described in [28]. Ward’s linkage clustering algorithm was used to identify clusters of individuals based on the four principal components. The gap statistic was used to determine the optimal number of clusters (if any), ranging from 1–7.

### Genomic validation of cell-based patient subtypes

CTh alterations are, in part, heritable [29–31] and relatively stable across the life-course in individuals with schizophrenia relative to other MRI-derived phenotypes [2], suggesting that genetic risk factors must be, to some extent, responsible for phenotypic CTh alterations observed in schizophrenia subjects. We thus aimed to validate cell-based patient subtypes with person-specific genomic data that capture genetic variation linked to changes in each neural cell type. For each individual, three separate polygenic scores were calculated for each patient cell-based subtype. Specifically, polygenic risk scores for schizophrenia (sczPRS) were constrained to gene sets using variants (i.e., single nucleotide polymorphisms [SNPs]) in and proximal to genes expressed within cell types relevant to a particular subtype. Supplementary Material presents detailed genotype and polygenic score calculation procedures.

To validate cell-based patient subtypes, partial correlations assessed the degree to which polygenic cell type scores covary with severity in whole-brain CTh deviations in individuals comprising the corresponding subtype. Correlation analyses accounted for the potential confounding effects of sex, genome-wide liability for schizophrenia (sczPRS) and familial relatedness (see Supplementary Material for genetic computation of familial relatedness). Discriminative validity was operationalized as endorsing the null hypothesis in non-subtype members. That is, CTh deviations in non-subtype members would not covary with genetic liability ascribed to matched cell types.

## Results

### CTh covaries with cell type patterned gene expression levels in healthy individuals

**Fig. 2:**
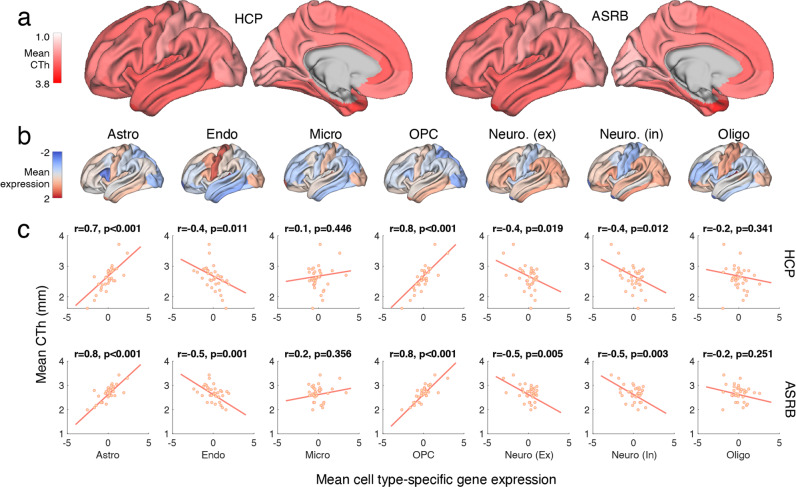
Cell type-specific gene expression maps and mean CTh variation in healthy controls. **a** Cortical renderings display mean Cortical thickness (CTh) for each region across all healthy individuals, respectively for HCP (discovery) and ASRB (validation) cohorts. **b** Cortical renderings display standardized (z-scores) for cell type-specific gene expression maps. **c** Scatter plots display associations between standardized cell type-specific gene expression maps and regional mean CTh in healthy controls. Each datapoint (34 in total) reflects a left hemispheric brain region from the Deskian-Killiany atlas, where mean CTh was computed across all healthy controls in the discovery or validation cohorts.

Consistent with previous work [32, 33], thicker brain regions were localized to multimodal cortical areas (e.g., the temporal pole) and the thinner regions comprised unimodal areas (e.g., the pericalcarine cortex). Regional CTh rankings were consistent across HCP (discovery) and ASRB (validation) cohorts (*r* = 0.953, *p* < 0.0001). Mean regional CTh across controls (Fig. 2a) was positively correlated to interregional expression levels of genes marking astrocytes and OPCs, and negatively correlated to expression patterns of genes enriched for endothelial cells, as well as for excitatory and inhibitory neurons (FDR*p* < 0.05; Fig. 2b). CTh variation did not significantly covary with interregional expression levels of gene groups for microglia or oligodendrocytes.

### Heterogeneity in individual CTh deviation profiles

**Fig. 3:**
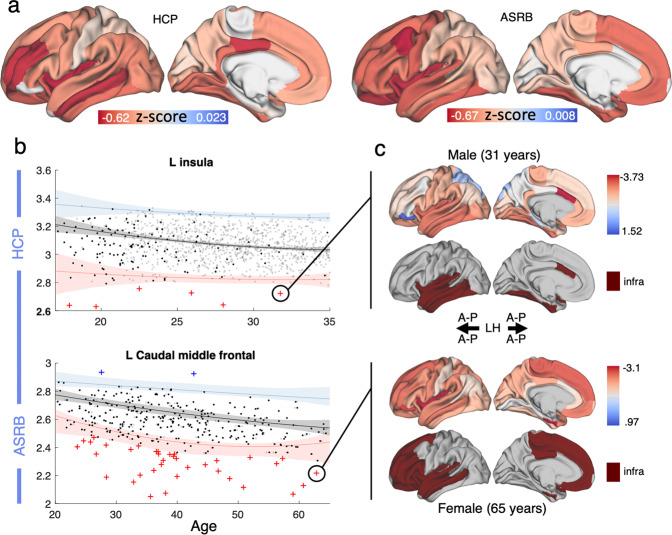
Deviation from normative ranges of CTh variation in individuals with schizophrenia. **a** Cortical renderings display the mean deviation score for each region across all individuals with schizophrenia, respectively for HCP and ASRB datasets. **b** Percentile curves for example regions. The 5th (red curve), 50th (black) and 95th (blue) percentiles quantify the range of variation among healthy individuals (dark dots) in the cortical thickness (CTh), as a function of age (horizontal axis) and sex. For each cortical region, individuals with schizophrenia were positioned on the normative percentile charts and then categorized as either: (i) *normal* (black); (ii) *supra-normal* (blue cross); or, (iii) *infra-normal* (red cross). The measurement unit of CTh is millimeters and age is quantified in years. Shading indicates 95% confidence intervals, estimated with bootstrapping (*n* = 1000). **c** Loci of variation in a 31-year old male (upper panel) and 65-year old female (lower panel) with schizophrenia.

To characterize CTh heterogeneity, normative ranges of variation in each brain region were established based on healthy individuals (*n* = 1127 for HCP [discovery] and *n* = 185 for ASRB [validation]) and operationalized as the range between 5% and 95% percentiles for a given age and sex (see Methods). For all cortical regions, more than 90% of the healthy individuals resided within the normative range, confirming the accuracy of the normative models. Figure 3a displays mean CTh deviation profiles across individuals with schizophrenia comprising both datasets.

The majority of individuals with schizophrenia (>80%) were within the normative CTh range established for each region. Individuals with schizophrenia showed more significant infra-normal deviations compared to controls, respectively for HCP and ASRB cohorts (Supplementary Figs. 3, 4). Across both HCP and ASRB cohorts, infra-normal deviations were most frequently located in frontal, temporal, and insular cortices and were most severe in the insula and rostral middle frontal cortex (HCP [discovery]; Fig. 3a) and caudal middle frontal gyrus (ASRB [validation]; Fig. 3a). At least one brain region with deviations from the established normative ranges was evident for most individuals with schizophrenia (discovery: 69%, validation: 72%), whereas only half of the healthy individuals showed significant regional variation within at least one brain region (healthy individuals in discovery cohort: 57%, *p* < 0.001; healthy individuals in validation cohort: 51%, *p* < 0.001). However, the regional loci of deviations were not consistent, with fewer than 20% of the individuals with schizophrenia showing significant deviations for any given region. As such, these findings cohere with previous normative modeling studies suggesting that regional variation in cortical gray matter abnormalities show substantial variation among individuals with schizophrenia [3, 13]. We next investigated whether this heterogeneity could reflect contributions of distinct cellular processes underlying CTh alterations across individuals with schizophrenia.

### Cell type-specific CTh alterations in schizophrenia

**Fig. 4:**
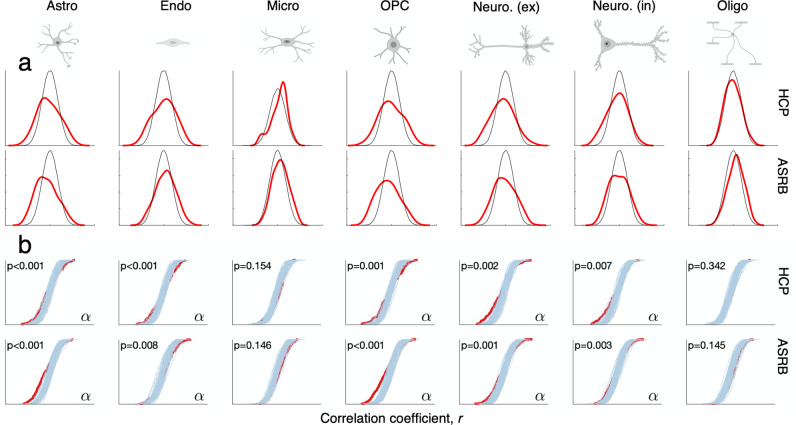
Association between cell type-specific gene expression patterns and CTh deviations in schizophrenia. **a** Density functions display the distribution of gene expression—CTh correlation coefficients (ranging from −1 to 1) for genes marking specific cell types. Plots display the null distribution of correlation coefficients (**black**), and the observed correlation coefficients (**red**) in patients comprising the HCP (discovery) and ASRB (validation) cohorts. **b** Plots display observed (**red**) and null cumulative distribution functions (**blue**) of correlation coefficients between regional gene expression and regional deviations in CTh. Results are presented for HCP (discovery) and ASRB (validation) cohorts. Alpha denotes a significant difference after FDR correction. These findings illustrate that the spatial patterning of CTh deviations significantly relate to expression gradients of genes that regulate specific cell classes. For example, the first plot shows that regional gene expression across genes marking astrocytes correlate more strongly with CTh deviation profiles in patients, relative to random deviation profiles (i.e., after permuting regional deviation values).

We examined associations between CTh deviation profiles of each schizophrenia subject with interregional gene expression levels of specific cell types (Fig. 4a). In the HCP (discovery) cohort, the observed distribution of interregional correlation coefficients was significantly more dispersed relative to the null distribution across five out of seven cell types (Ansari-Bradley FDR < 0.05, Fig. 4b). Differences from the null distribution were characterized by stronger negative correlations, meaning that CTh losses covaried with higher cell-type gene expression in schizophrenia. These findings were reproduced in the ASRB (validation) cohort, where the observed distribution of regional correlation coefficients was significantly more dispersed than a null distribution in the five same cell types. As such, CTh deviations relate more strongly to interregional cell type gene expression levels in *some* individuals with schizophrenia, than can be attributed to chance alone.

### Characterizing cell-based patient subtypes

Unsupervised clustering of interregional CTh-cell type gene expression associations distinguished three subtypes based on the gap criterion. In the discovery cohort (Supplementary Fig. 5), Subtype I comprised 26% of patients displaying regional cortical thinning aligned to high neuronal, endothelial, and oligodendrocyte gene expression. Subtype II comprised 27% of patients marked by regional cortical thinning that was weakly related to neuronal cell types. Finally, subtype III comprised 47% of patients characterized by regional cortical thinning in regions with high gene expression of astrocytes, and OPCs.

**Fig. 5:**
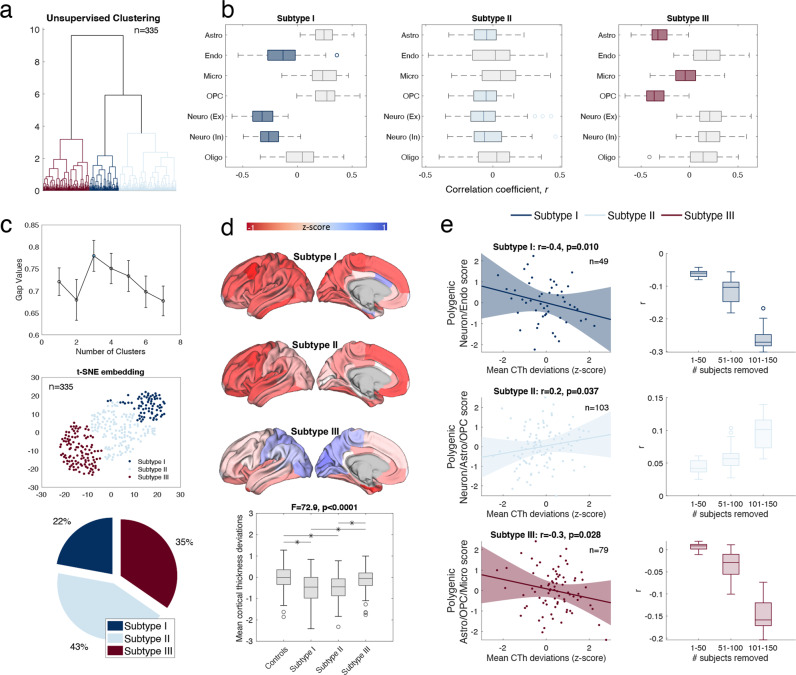
Cell-based patient subtypes and genomic validation. Results are shown for the validation (ASRB) cohort **a** Person-specific correlations between cortical deviations with interregional cell type-specific gene expression maps were clustered into three cell-based subtypes. **b** Boxplots show characteristic gene expression-CTh deviation association patterns for each subtype. Colored boxes denote negative associations (i.e., where CTh loss maps onto higher cell type gene expression) that significantly differ from zero (pFDR < 0.05). Box edges indicate 25th and 75th percentiles of inter-individual variation in standardized gene expression for each cell type. Central mark indicates median, whisker extend to the most extreme datapoints, and circles denote outliers. **c** The top line plot demonstrates that the maximum gap criterion occurs at three clusters, which is more than one standard error from the next maximum gap value. The bottom plot shows the t-Distributed Stochastic Neighbor Embedding (t-SNE), whereby person-specific points are embedded into three clusters in a way that respects similarities between points. The pie chart displays the portion of schizophrenia subjects comprising each subtype. **d** Brain renderings display mean CTh deviation profiles across individuals from each subtype, respectively. The boxplot displays mean cortical thickness deviations across the three subtypes (bars = median, boxes = lower and upper quartiles and circles = outliers computed from the interquartile range). **e** Scatterplots (left column) present correlations between polygenic scores (*y*-axes) and cortical thickness deviations (*x*-axes), colored according to cell-based subtype (e.g., dark blue datapoints represent individuals comprising Subtype I-Neuronal). Shaded areas represent the 95% confidence interval, colored according to subtype. Boxplots (right column) display r values for correlations evaluated using schizophrenia subjects sorted by subtype membership (ranging from zero to one). For example, ‘Soft Subtype I’ shows that correlation strength increases as a function of membership to Subtype I—i.e., the r value decreases as more subjects with low membership scores are excluded from the correlation analyses.

Consistent with the discovery cohort, a three-cluster solution was achieved in the validation cohort (Fig. 5a–c). Subtype I comprised 22% of patients displaying regional cortical thinning aligned to high neuronal and endothelial (but not oligodendrocyte) gene expression. Subtype II comprised 43% of patients marked by regional cortical thinning that weakly relates to neuronal, astrocytes, and OPCs. Finally, subtype III comprised 25% of patients characterized by regional cortical thinning in regions with high gene expression of astrocytes, OPCs, and microglia, albeit to a lesser extent. Cell type-CTh deviation associations were statistically conserved between the cohorts in Subtype I (*r* = 0.97, *p* = 4.05e−04) and Subtype III (*r* = 0.99, *p* = 1.12e−05), and nominally conserved in Subtype II (*r* = 0.70, *p* = 0.04), which comprised weaker cell type associations with CTh deviations in both cohorts.

The broad cell-class stratification was not explained by demographic (age and sex) or clinical factors (illness duration, positive symptoms, negative symptoms; Supplementary Table 6). As shown in Fig. 5d, Subtypes I and II were characterized by widespread cortical thinning, in contrast to Subtype III, which showed comparatively milder fronto-temporal thinning, as well as preserved occipital CTh. A similar pattern emerged at the whole-brain level, whereby mean CTh deviation scores computed across the entire brain were lowest in individuals comprising Subtype I and highest in those residing in Subtype III (Fig. 5d).

### Genomic validation of cell-based patient subtypes

Genomic validation of cell-based patient subtypes was performed in the ASRB (validation) cohort, where genotype data were available for 231 individuals with schizophrenia (70 females, 161 males). Cell type-specific polygenic scores are shown in Supplementary Table 7. Polygenic scores were averaged across cell type gene sets to yield one polygenic score relevant to each subtype in the validation cohort, quantified as negative correlations that significantly differ from zero (FDR*p* < 0.05; Fig. 5a). Negative correlations denote cell types for which higher gene expression mapped onto infra-normal CTh deviations (i.e., CTh losses). Therefore, the validation results, presented in turn, tested whether individuals with higher polygenic risk ascribed to specific cell types display more pronounced cortical thinning in regions linked to higher gene expression of matching cell types.

As expected, higher polygenic neuronal/endothelial scores significantly covaried with lower mean CTh deviation scores (i.e., cortical thinning) in individuals comprising Subtype I (Fig. 5c; *n* = 49, *r* = −0.40, FDR*p* = 0.010). Importantly, this association was not significant in Subtype II or Subtype III (p > 0.05; Supplementary Fig. 6), confirming the specificity of our result to individuals residing in Subtype I and the discriminant validity of cell-based subtypes. Regarding Subtype II, a significant positive correlation was seen between mean CTh deviation scores with summary polygenic scores constrained to genes marking neuronal cells, astrocytes and OPCs (*r* = 0.20, FDR*p* = 0.037). This relationship was not significant in individuals within Subtype I or III. Finally, a significant negative correlation was seen between mean CTh deviation scores with polygenic scores constrained to genes marking astrocytes and OPCs in individuals comprising Subtype III (*r* = −0.30, FDR*p* = 0.028) but not in Subtype I or II. Subtype-specific relationships were replicated using an alternative liberal genic boundary to define polygenic scores (Supplementary Fig. 7).

Although three subtypes provided the optimal clustering solution, they were not well differentiated (see Fig. 5c), suggesting that the characteristics of cell-based patient subtypes are not mutually exclusive. Rather, individuals differ in the degree to which they exhibit cell type-patterned deviations. Therefore, we additionally validated subtypes based on ‘soft’ cluster assignments, whereby individual membership of each subtype was characterized continuously from zero to one to capture within-cluster variation. As shown in Fig. 5e (left column), the correlation strength between polygenic neuronal/endothelial scores and mean CTh deviation scores increased as a function of subtype membership. This effect was repeated in Subtypes II and III, suggesting that severity of cell patterned CTh deviations covary with a portion of genetic liability ascribed to matched cell types.

## Discussion

We systematically combined information across three biological scales—genes, gene expression, and brain morphometry—to understand possible sources of CTh heterogeneity in schizophrenia. Bridging gaps across levels of biological organization is a necessary step toward uncovering realistic therapeutic strategies, which target molecules and cells that in turn elicit responses in whole brain circuits.

The current investigation is part of a growing effort to parse neuroimaging-derived phenotypic heterogeneity into more homogeneous subgroups (e.g., [34–40]) with a view to expose disease mechanisms, identify meaningful biological differences between individuals, and to open avenues for targeted investigations and interventions in psychiatry [41, 42]. Here, we show that CTh heterogeneity in schizophrenia relates to cellular processes deduced from whole-brain transcriptomic and person-specific genomic data. Across two independent cohorts, CTh deviations significantly covaried with interregional expression levels of gene sets marking astrocytes, endothelial cells, OPCs, and excitatory and inhibitory neuronal cells. However, substantial heterogeneity was evident: about half of all patients had a genetic load related to glial cells and oligodendrocyte progenitors and they displayed less severe cortical thinning. Approximately a third had mixed genetic loading and widespread cortical thinning, and approximately a fifth had neuronal loading combined with widespread thinning. Therefore, schizophrenia subjects displayed spatially patterned CTh deviations that differentially aligned with distinct cell types. This cell-based stratification was validated against patient-specific genetic variation by leveraging schizophrenia risk alleles enriched in cellular processes.

Our finding of multiple cell type contributions to schizophrenia CTh alterations is consistent with a previous transcriptome-MRI association study [8]. Here, we extend these findings to delineate neural correlates of person-specific CTh alterations, enabling stratification of patients into cell-based subtypes. Our results are also compatible with molecular evidence that transcriptomic alterations display cell-type specific features [43]. For example, recent postmortem studies that isolate cell specific transcriptomic changes revealed 1400 differentially expressed genes in layer III and V pyramidal cells [44] and 800 differentially expressed transcripts in Layer III parvalbumin-containing interneurons [45] within the dorsolateral prefrontal cortex (DLPFC) of schizophrenia-affected brains.

We present an approach to non-invasively track person- and cell type-specific phenotypes in vivo. Individuals with schizophrenia were stratified into three cellular subtypes based on regional CTh deviations from normative ranges of variation established in healthy comparison individuals of the same age and sex. Each subtype manifested a characteristic CTh deviation profile that spatially aligned with transcriptomic expression marking specific cell types. This result does not negate probable roles for multicellular pathologies within individuals with schizophrenia. Rather, we contend that spatially aligned expression data can expose unique CTh deviation patterns that converge on specific cell type/s. Stratification by dominant cell types can in turn facilitate neurobiological interpretation of heterogeneous CTh deviation profiles in schizophrenia and other psychiatric/neurological disorders. For example, CTh alterations characteristic of Subtype I may underlie alterations in neuronal morphology (e.g., reduced neuropil) or cell density (e.g., reduced excitatory pyramidal or GABAergic interneuron populations) observed in neuropathological studies of schizophrenia-affected brains [11]. While the present study cannot infer specific mechanisms, a cell-based stratification of individuals with schizophrenia narrows the search space for potential mechanisms that drive overt signatures of disease within each subtype.

What makes an integrated transcriptomic-imaging approach appealing is the ability to infer biological relevance from in vivo MRI data; however, caution is warranted against over-interpreting these findings in isolation. Specifically, spatially resolved gene expression data derive from externally sourced healthy adult donors and thus, cannot precisely capture interregional expression patterns in the context of other individuals, particularly in a disease state. To address this challenge, we demonstrate that in vivo cell-based subtypes can effectively be integrated with person-specific genomic data, making validation possible. Using this approach, we observed a polygenic contribution to CTh alterations presenting in a subtype-specific manner. These findings provide crucial links between genome, transcriptome, and MRI-derived phenotypes, demonstrating the feasibility of integrating information across multiple biological scales.

Several limitations require consideration. First, regional nonuniformity in cell type-specific gene expression may depend on cell numbers and/or single-cell transcription levels. While postmortem evidence across four species demonstrates that thicker regions contain more glial cells [32], nonuniformity in terms of gene regulation of specific cells remains unclear due to difficulties in measuring single-cell transcription levels. Second, patterns of CTh alterations that do not map neatly onto specific cell types may weaken the predictive power of polygenic risk. This may explain only nominally significant validation results in the mixed-cell subtype. Third, cell type associations with MRI-derived phenotypes hinge on the estimation of cell type transcriptional maps. For example, Shin et al. [46] observed a positive correlation with transcriptional maps of neurons and microglia in healthy controls. This contrasts with our study, which observed a negative correlation with neuronal maps and no correlation with microglia in healthy controls. The major difference between Shin et al. [46] and our approach is the source data used to extrapolate gene sets. More specifically, Shin et al. [46] selected gene sets based on single-cell RNA extracted from mouse brain tissue [47], whereas our gene sets were defined on single-cell studies of the adult human cortex [20].

## Conclusions

We classified two independent schizophrenia cohorts into cell-based subtypes based on individual CTh deviation profiles and established gene expression data. This multiscale—imaging and transcriptomic—stratification relates to polygenic liability in a cell type-specific manner. Therefore, while schizophrenia is a multicellular disease, the composition and degree of cell type-specific involvement likely differs across individuals. Improving cell type-specific characterization in schizophrenia facilitates neurobiological interpretation of large-scale MRI phenotypes and expands our understanding of heterogeneity in the disorder. We envisage that cell-based stratifications based on MRI phenotypic and genomic information will guide future framing of hypotheses and improve the clinical relevance and predictive power of in vitro disease models.

## Supplementary information

Supplementary Material

## Data Availability

Genetic, clinical, and brain imaging data can be requested from the Australian Schizophrenia Research Bank (https://www.neura.edu.au/discovery-portal/asrb/). Brain imaging and demographic data from the Human Connectome Project-Young Adult (S1200) can be requested from https://www.humanconnectome.org/study/hcp-young-adult/overview. Brain imaging and clinical data comprising the Human Connectome Project-Psychosis dataset can be requested from https://www.humanconnectome.org/study/human-connectome-project-for-early-psychosis. Access to all three datasets is subject to approval. All relevant data to generate cell type transcriptional maps can be found here: https://github.com/jms290/PolySyn_MSNs. Gene lists can be downloaded directly from https://static-content.springer.com/esm/art%3A10.1038%2Fs41467-020-17051-5/MediaObjects/41467_2020_17051_MOESM8_ESM.xlsx

## Code availability

The Matlab function quantreg was used to perform quantile regression with bootstrapping confidence intervals. The Matlab function evalclusters was used to determine the optimal cluster solution, and linkage and cluster functions determined cluster membership. The code to generate cell type gene expression maps can be downloaded from https://github.com/RafaelRomeroGarcia/geneExpression_Repository.
