# Supplementary material for: Cell type-specific manifestations of cortical thickness heterogeneity in schizophrenia
Source: Mol Psychiatry. 2022 Feb 10;27(4):2052–60. doi: 10.1038/s41380-022-01460-7 (PMC9126812; doi:10.1038/s41380-022-01460-7)
Supplement: Supplementary file 1 — Supplementary Material [file 41380_2022_1460_MOESM1_ESM.pdf]

Supplementary Material

Table of Contents

TABLE 1. SITE AND CLINICAL CHARACTERISTICS – HCP (DISCOVERY) COHORT 2

TABLE 2. SITE AND CLINICAL CHARACTERISTICS – ASRB (VALIDATION) COHORT 3

MRI ACQUISITION, PROCESSING, AND HARMONIZATION 4

SUPPLEMENTARY FIGURE 1. HARMONIZATION. 5

ESTABLISHING NORMATIVE RANGES WITH QUANTILE REGRESSION. 6

MAPPING CELL TYPE-SPECIFIC GENE EXPRESSION PATTERNS IN THE HUMAN BRAIN. 6

SCHIZOPHRENIA GENOME-WIDE ASSOCIATION DATA. 7

GENE SET ASSOCIATION OF CELL-TYPE GENE LISTS. 7

SUPPLEMENTARY TABLE 3. GENE-SET ASSOCIATION OF SEVEN CELL-TYPE GENE-SETS WITH SCHIZOPHRENIA 8

SUPPLEMENTARY TABLE 4. NEURONAL GENE-SET ASSOCIATION COVARIED FOR CORTICAL GENE EXPRESSION 8

SUPPLEMENTARY TABLE 5. NEURONAL GENE-SET ASSOCIATION RESULTS FOR CLOZUK+PGC2 META-ANALYSIS 8

SUPPLEMENTARY FIGURE 2. GENE-SET ASSOCIATION OF CELL-TYPE SPECIFIC GENE-SETS WITH SCHIZOPHRENIA. 9

GENOTYPING AND POLYGENIC RISK SCORES. 10

SUPPLEMENTARY FIGURE 4. PERCENTAGE OF INDIVIDUALS RESIDING OUTSIDE THE NORMATIVE RANGE IN REGIONAL CORTICAL THICKNESS. 11

SUPPLEMENTARY FIGURE 5. DISTRIBUTION OF CORTICAL LOCI WITH DEVIATIONS. 12

SUPPLEMENTARY FIGURE 6. CELL-BASED PATIENT SUBTYPES IN THE DISCOVERY (HCP) COHORT. 13

ILLNESS FACTORS AND BROAD CELL-TYPE STRATIFICATION. 14

SUPPLEMENTARY TABLE 6. DEMOGRAPHIC AND ILLNESS FACTORS GROUPED BY BROAD CELLULAR SUBTYPE 14

SUPPLEMENTARY TABLE 7: SUMMARY OF RAW PRS FOR EACH GWAS AND SUBSET OF GENES 15

SUPPLEMENTARY FIGURE 7. SPECIFICITY ANALYSES. 16

SUPPLEMENTARY FIGURE 8. VALIDATION RESULTS USING A LIBERAL GENIC BOUNDARY. 17

Table 1. Site and clinical characteristics – HCP (Discovery) cohort

	HCP (Discovery)		
	HCP-Psychosis		HCP-1200
	SZ (n=140)	HC (n=62)	HC (n=1065)
**Demographics**			
Age, y mean (SD)	22.782 (3.83)	24.30 (4.10)	28.75 (3.67)
Sex, n (%) Females	48 (34)	23 (37)	575 (54)
IQ (WASI), mean (SD)	102.55 (16.89)	115.36 (11.04)	
**Site**	**n (%)**		
Brigham and Women's Hospital	43 (21)	30 (15)	
Indiana Hospital	60 (30)	21 (10)	
McLean Hospital	37 (18)	11 (5)	
**Clinical Characteristics**	**mean (SD)**		
Illness Duration, *y*	1.83 (1.35)	N/A	
Positive symptoms (PANSS)	13.75 (4.98)	N/A	
Negative Symptoms (PANSS)	12.40 (5.12)	N/A	
General psychopathology (PANSS)	48.67 (11.28)	N/A	
**Medication**			
Current antipsychotic CPZ-eq dose	360.53 (1000)	N/A	
Antipsychotic exposure duration, *mo*	17.13 (15.47)		

Abbreviations: Schizophrenia (SZ), healthy control (HC), years (y), months (mo), Positive and Negative Syndrome Scale (PANSS), Chlorpromazine-equivalent (CPZ-eq).

Note. A normative modeling approach reduces the need for sex and aged-matched groups given that percentiles are first defined on healthy controls as a function of age and sex, and then each individual patient is referenced to the resulting normative range. For this approach, it is important that the training group (i.e., healthy controls) spans the age range of the individuals to which the normative model will be applied, but precise matching is not needed.

Table 2. Site and clinical characteristics – ASRB (Validation) cohort

	ASRB (Validation)	
	**SZ** (n=335)	**HC** (n=185)
**Demographics**		
Age, y mean (SD)	39.70 (10.81)	41.05 (14.02)
Sex, n (%) Females	100 (30)	94 (51)
IQ (WASI)	104.77 (15.28)	116.86 (11.00)
**Site**	**n (%)**	
Sydney	69 (13)	38 (7)
Melbourne	89 (17)	71 (14)
Perth	38 (7)	26 (5)
Brisbane	112 (22)	34 (7)
Newcastle	27 (5)	16 (3)
**Clinical Characteristics (DIP)**	**mean (SD)**	
Illness Duration, *y*	15.74 (9.83)	N/A
Positive symptoms	7.69 (3.70)	N/A
Negative Symptoms	25.80 (18.95)	N/A
Global functioning	54.49 (13.77)	N/A
**Medication**	**n (%)**	
Any antipsychotic	252 (75)	N/A
Typical antipsychotic	235 (70)	N/A
Atypical antipsychotic	33 (10)	N/A
Antidepressant	100 (30)	N/A
Anticonvulsant/Mood stabilizer	43 (13)	N/A
Anti-cholinergic	11 (3)	N/A
Anxiolytic	35 (10)	N/A
Missing	86 (26)	N/A

Abbreviations: Schizophrenia (SZ), healthy control (HC), years (y), Diagnostic Interview for Psychosis (past year)

MRI acquisition, processing, and harmonization.

Acquisition. T1-weighted brain MRI scans were acquired as follows: HCP-1200 (3T Connectome Siemens Skyra: Voxel resolution=.7mm^3, TR=2400ms, TE=2.14ms); HCP-Psychosis (Siemens MAGNETOM Prisma 3T: Voxel resolution=.8mm^3, TR=2400ms, TE=2.22ms); and ASRB (Siemens Avanto 1.5T: Voxel resolution=1mm^3, TR=1980ms, TE=4.3ms).

Processing. Image preprocessing, quality control procedures and cortical measurement estimation was previously performed for the HCP-1200 [1] and ASRB datasets [2, 3]. T1-weighted images comprising the ASRB dataset were processed using FreeSurfer version 6 [4]. Processing and CTh estimation for the HCP-psychosis dataset followed the HCP-1200 pipeline to facilitate harmonization of the HCP datasets. In brief, T1-weighted images for these datasets were processed using FreeSurfer version 5.3 [4]. For all three datasets, preprocessing included intensity normalization, non-brain tissue removal and spatial normalization to Talairach-like space. Preprocessed images were segmented into gray matter, white matter and cerebrospinal fluid, after which white matter and gray matter surfaces were reconstructed. CTh was estimated for each surface vertex based on the distance separating the gray and white matter surfaces and averaged over all vertices comprising each of the 68 cortical regions (34 per hemisphere) of the Desikan-Killiany atlas [5, 6].

Quality control. For the HCP-psychosis cohort, where quality control was not previously performed, raw data were checked for anatomical, processing and noise issues. Furthermore, FreeSufer quality control was performed as per ENIGMA protocols http://enigma.ini.usc.edu/protocols/imaging-protocols/, which generates medial and lateral snapshots for visual inspection of FreeSurfer reconstructions. All images satisfied reconstruction criteria (i.e., all lobes were present and label positions did not differ from the general case). For the HCP-1200 cohort, subjects with documented brain anatomical, processing or noise issues (n=151) based on the HCP supplied 'QC_Issue' variable were excluded from the current analyses.

Harmonization. Regional CTh estimates were retrospectively harmonized with ComBat [7] to alleviate possible site-related differences, while retaining biological variation related to diagnosis, sex and age. For HCP (discovery), each measure was harmonized across 4 sites: HCP-young adult sample (site 1) and 3 sites comprising the HCP-Psychosis dataset. For ASRB (validation), each measure was harmonized across 5 sites comprising the ASRB dataset. Harmonization successfully removed all scanner differences (see Supplementary Figure 1).

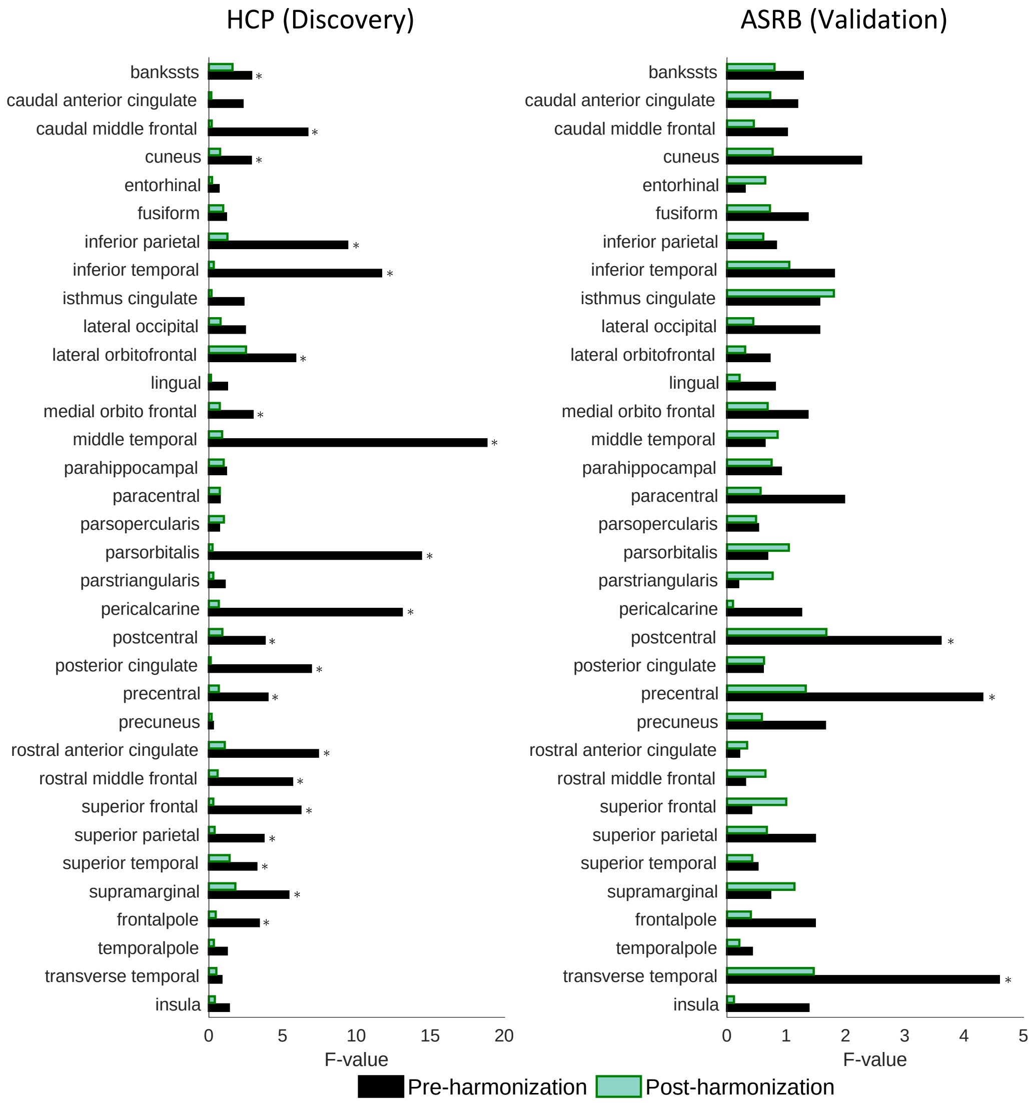

Supplementary Figure 1. Harmonization. Between-scanner differences in regional cortical thickness estimates were examined pre and post harmonization (using ComBat) by comparing healthy controls from the separate scanners comprising the HCP (discovery; see Supplementary Table 2) and ASRB (validation; see Supplementary Table 3) cohorts. Bars show the f-value obtained from Analyses of Covariance (ANCOVA), with regional cortical thickness measures as dependent variables and age and sex as covariates. Asterisks' denote significant differences in cortical thickness estimates between the scanners (p<.05, uncorrected). Harmonization successfully removed all scanner differences in regional cortical thickness estimates.

Establishing normative ranges with quantile regression.

Quantile regression [8] was used to obtain a normative range of regional CTh variation [2] based on the following model,

$$CTh_i = \beta_0 + \mathrm{sex}_i\, \beta_1 + (\mathrm{age}_i - A)\, \beta_2 + \mathrm{sex}_i\, (\mathrm{age}_i - A)\, \beta_3 + (\mathrm{age}_i - A)^2\, \beta_4 \qquad (1)$$

For the participant with index i, sex_i encodes the participant's sex (male: 0, female: 1), age_i is the participant's age. Under this model, β_0 is the estimated regional CTh for a male of age A, β_1 is the between-sex difference in CTh at age A, β_2 is the rate at which CTh changes per year at age A, while β_3 and β_4 model the sex-by-age interaction and quadratic age effects, respectively. Bootstrapping was used to estimate confidence intervals for the 5% and 95% percentiles of regional CTh variation.

Mapping cell type-specific gene expression patterns in the human brain.

Alignment of anatomically resolved gene expression data to the left hemisphere of the Desikan-Killiany atlas parcellation was undertaken previously (https://github.com/RafaelRomeroGarcia/geneExpression_Repository), as described elsewhere [9-11]. We previously confirmed the generalizability of gene expression profiles across the six donors in the AHBA in a leave-one-donor-out cross validation procedure, suggesting that anatomical gene expression patterns are broadly conserved across the six adult brains [9]. In brief, the T1-weighted brain MRI scans for each AHBA postmortem brain was first parcellated using FreeSurfer. Tissue samples were assigned to the nearest parcel centroid of left hemispheric Desikan-Killiany regions. For the two postmortem brains with only right hemisphere data, tissue samples were first projected onto the left hemisphere before aligning the data to the atlas. Median regional expression was estimated for each gene across participants (N=6) [9]. Then, genes were assigned to seven specific cell types using gene sets from large-scale single-cell studies of the adult human cortex [12-16]. Mean expression of each cell-type gene set was computed in each of the 34 (left hemisphere) cortical regions and normalized (converted into z-scores) by mean expression across the entire brain.

Schizophrenia genome-wide association data.

Schizophrenia genome-wide association study (GWAS) summary statistics were obtained from the psychiatric genomics consortium (PGC) for 161405 participants of predominantly European ancestry as described extensively elsewhere [17]. A smaller, European ancestry GWAS meta-analysis of PGC and CLOZUK samples (N=105318) was also utilised as a sensitivity analysis [18].

Gene set association of cell-type gene lists.

Linear regression models were constructed to test associations between schizophrenia and the seven gene-sets representing expression in the different cell types using MAGMA v1.09[19]. Briefly, MAGMA aggregates SNP-wise *P* values at gene-level to act as the unit of effect in a test of gene-set association. Gene-based P values were calculated using the snp-wise=mean MAGMA model, whereby the test statistic is the sum of squared SNP-wise Z scores, with Z ~ $N(0,S)$, given S is a matrix of correlations between SNPs based on estimates of linkage disequilibrium (LD). Autosomal gene coordinates in hg19 assembly were obtained from NCBI and the 1000 genomes phase 3 European panel was utilized as an LD reference. Genes within the major histocompatibility complex (MHC) region were not considered due to the haplotype complexity of that region, as is usual practice [20, 21]. We extended the boundaries of the genic coordinates upstream and downstream to capture regulatory variation with both a conservative (5 kilobases (kb) upstream, 1.5 kb downstream) and liberal genic (35 kb upstream, 10 kb downstream) boundary definition used for analyses.

After probit transformation of gene-based *P* to *Z*, a linear regression model was constructed such that *Z* was the outcome and a binary indicator of gene-set membership to test whether genes in the set were more associated than all other genes considered. This model was covaried for confounders including gene-size and minor allele count [19, 22]. We used the Bonferroni method to correct for multiple comparisons, with the threshold set to $P < 3.57 \times 10^{-3}$ to account for the seven (cell type) tests using two different genic boundary configurations. Given that the gene-sets considered are highly brain expressed, we additionally constructed a model covaried for the cortical expression of each gene (median transcript per million) to assess whether this may bias the estimated association [20, 23].

Results. Neuron-linked genes were strongly associated with schizophrenia irrespective of a conservative or liberal genic boundary definition (Supplementary Table 3 and Supplementary Figure 2). Covariation for cortical gene-expression resulted in only a minor effect on the estimated coefficient, supporting that the relationship between these sets and schizophrenia is not simply driven by high neurological expression of these genes (Supplementary Table 4). Moreover, gene-set association utilising a previous, smaller schizophrenia GWAS also replicated the association of these two gene-sets (Supplementary Table 5). The oligodendrocyte gene-set also survived Bonferroni correction with liberal or conservative boundary definitions (Supplementary Table 3), however, these were comparatively weaker signals. Nominal association ($p<0.05$) was also observed for the oligodendrocyte precursor set with a conservative genic boundary configuration; however, this was not significant after correction for either genic boundary definition (Supplementary Table

3). None of the remaining sets (astrocyte, endothelial cells, and microglia) displayed evidence of association with schizophrenia.

Supplementary Table 3. Gene-set association of seven cell-type gene-sets with schizophrenia

SET	BETA	SE	P	BOUNDARIES
Astro	0.005	0.041	0.45289	Conservative
Endo	-0.138	0.036	0.99993	Conservative
Micro	-0.131	0.042	0.99899	Conservative
Neuro-Ex	0.244	0.036	7.814E-12	Conservative
Neuro-In	0.277	0.044	1.08E-10	Conservative
Oligo	0.069	0.040	4.34E-02	Conservative
OPC	0.182	0.106	0.042391	Conservative
Astro	0.038	0.045	0.19898	Liberal
Endo	-0.143	0.040	0.99981	Liberal
Micro	-0.094	0.046	0.97992	Liberal
Neuro-Ex	0.255	0.038	1.551E-11	Liberal
Neuro-In	0.328	0.045	2.66E-13	Liberal
Oligo	0.129	0.043	1.28E-03	Liberal
OPC	0.178	0.110	0.052686	Liberal

[1]Conservative refers to the genic boundaries for variant to gene annotation, that is, gene coordinates were extended 5 kb upstream and 1.5 kb downstream to capture regulatory variation, [2]Liberal genic boundaries were 35 kb upstream and 10 kb downstream.

Supplementary Table 4. Neuronal gene-set association covaried for cortical gene expression

SET	NGENES	BETA	BETA SD	SE	P	BOUNDARY
Neuro-Ex	1097	0.238	0.058	0.037	3.82E-11	Conservative
Neuro-In	788	0.273	0.057	0.044	3.12E-10	Conservative
Neuro-Ex	1097	0.255	0.062	0.039	2.25E-11	Liberal
Neuro-In	788	0.320	0.066	0.046	1.36E-12	Liberal

Supplementary Table 5. Neuronal gene-set association results for CLOZUK+PGC2 meta-analysis

SET	BETA	SE	P	BOUNDARIES
Neuro-Ex	0.22	0.04	3.50E-10	Conservative
Neuro-In	0.25	0.04	2.60E-09	Conservative
Neuro-Ex	0.21	0.05	7.50E-09	Liberal
Neuro-In	0.25	0.02	2.70E-09	Liberal

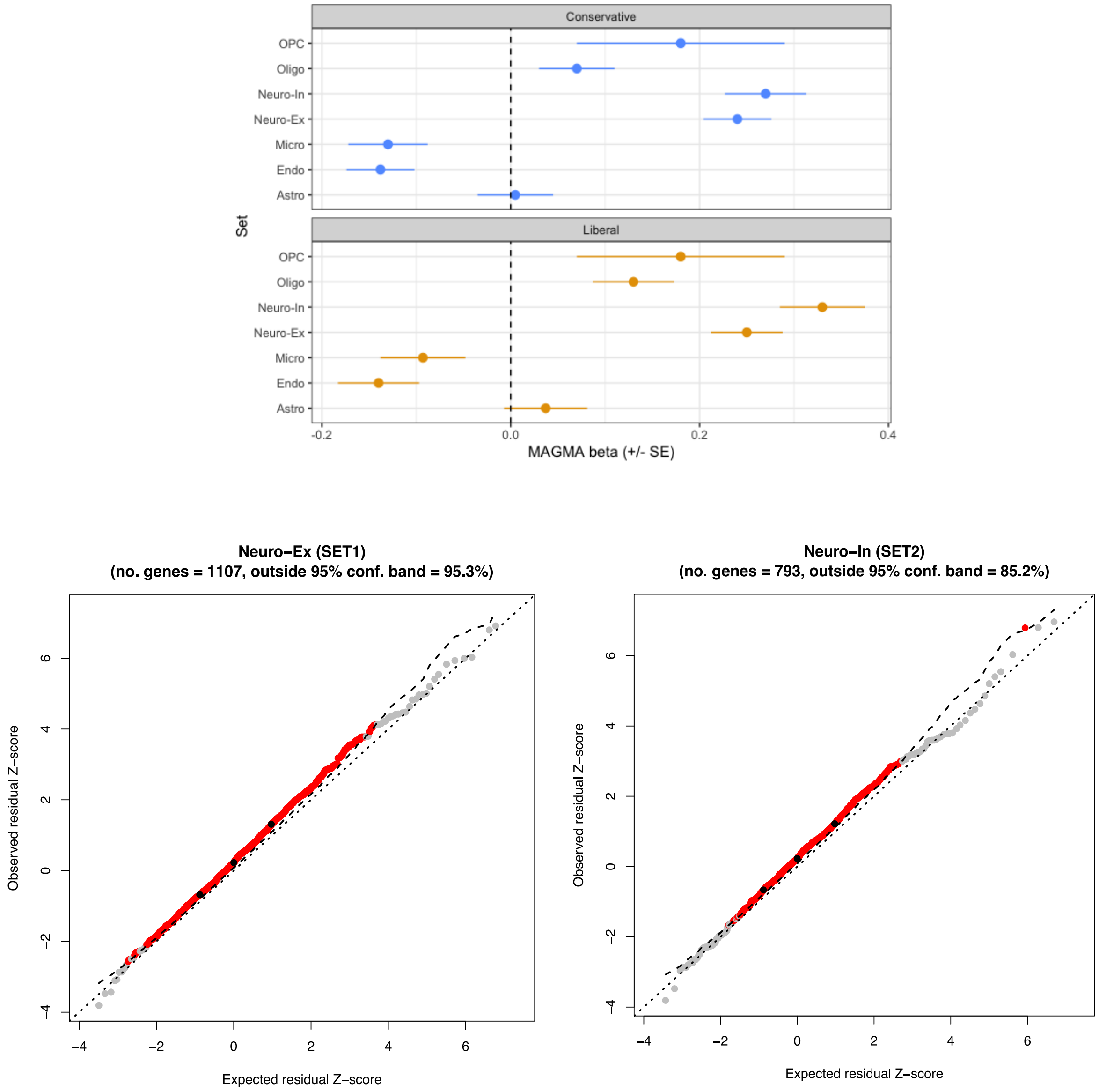

Supplementary Figure 2. Gene-set association of cell-type specific gene-sets with schizophrenia. ***(a)*** *Forest plot of MAGMA gene-set association results for seven cell-type gene-sets: OPC = oligodendrocyte precursors, Oligo = oligodendrocyte, Neuro-In = inhibitory neurons, Neuro-Ex = excitatory neurons, Micro = microglia, Endo = endothelial cells, Astro = astrocytes. Each panel displays the results constructing a model using liberal or conservative definitions the boundaries for each gene. Conservative boundaries extend the gene 5 kb upstream and 1.5 kb downstream to capture regulatory variation, whilst liberal boundaries are 35 kb and 10 kb upstream and downstream, respectively. The MAGMA beta-coefficients for the gene-set term are plotted with the error bars representing the standard error of the coefficient.* ***(b)*** *QQ-plots of residualised genic Z values from a null model without the gene-set term for the inhibitory and excitatory neuron genesets (conservative boundaries). Black points denote the 25th, 50th, and 75th quantiles, whilst the dotted line is the one-sided, upper 95% confidence band of deviation from the diagonal by chance alone. Points are shaded red to indicate if they fall above the confidence band or grey otherwise. As shown, Z scores deviated reasonably early and consistently in the set from the plot diagonal, with the majority of observed residual Z values outside the upper 95% confidence band of deviation expected by chance alone. Points are shaded red to indicate if they fall above the confidence band or grey otherwise. As shown, the association of the excitatory and inhibitory sets with schizophrenia was not driven by only a subset of genes in the set [23]. Z scores deviated reasonably early and consistently in the set from the plot diagonal, with the majority of observed residual Z values outside the upper 95% confidence band of deviation expected by chance alone. It should be noted that approximately 13% and 20% of the genes in the excitatory and inhibitory sets, respectively, do not surpass the 95% confidence band, which suggests some non-uniformity in the genic association across the sets. However, given these are large gene-sets over 700 genes each it is unlikely that any single overlapping gene-set would be responsible for the observed associations.*

Genotyping and polygenic risk scores.

Genotype data was originally performed using Illumina 610K genotyping arrays imputed to the 1000 genomes phase 3 European reference panel and was filtered for variants with a minor allele frequency (MAF) > 1%, Hardy-Weinberg equilibrium exact test p-values > $1x10^{-6}$, and call rates > 2%; all individuals further passed a variant missingness threshold of 2%. We selected unrelated, European ancestry individuals, as described previously [24, 25]. Briefly, physically genotyped, autosomal SNPs (MAF > 0.05) in relative linkage equilibrium (R2 < 0.1) were input for relatedness testing, whilst regions of long-range linkage disequilibrium were excluded. Individuals with high relatedness defined by genome wide identity by state (pi_hat > 0.185) were removed. Principal components analysis (PCA) was undertaken with PLINK 1.9. The first three SNP derived principal components quantified the familial relatedness confound for validation analyses. Population outliers were excluded using *k*-means clustering, wherein the five generated clusters represented the super-populations in the 1000 Genomes Phase 3 reference panel. Eigenvectors were then derived in the same fashion for the European subset to use as covariates for population stratification in downstream analyses.

For each individual, polygenic risk scores (PRS) were calculated based on the PGC summary statistics [17] using PRSice v2.3.3 [26]. Variants were selected using a P value threshold of 0.05 – that is, only variants with a nominally significant association (P < 0.05) contributed to the final score. This value was selected based on the optimal p-value threshold determined in the respective publications for each GWAS [27, 28]. A genome wide PRS was calculated based on all genotyped variants present within the respective GWAS data. Additionally, for each individual, seven separate scores were calculated based on variants in and proximal to genes expressed within each cell type, based on the same gene sets used to generate cell type-specific gene expression maps and as derived from single-cell sequencing of adult human brain tissue [12-16]. Both liberal and conservative genic boundaries were defined, as above for the gene-set association models. The genome-wide and gene-set PRS were calculated assuming an additive model (equation 2), whereby the estimated effect size for *j* variants ($\hat{\beta}$) is multiplied by their dosage in individual *i* (G).

$$PRS_j = \sum_i \hat{\beta}_j G_{ij} \quad (2)$$

The resulting score was divided by the number of alleles for the jth individual, which is the default PRSice model. To validate subtypes with polygenic cell scores, Neuro-Ex and Neuro-In genes were combined to yield one polygenic neuron score; Astro and OPC genes were combined to yield one Astro/OPC score and all seven-cell type polygenic scores were combined to yield one polygenic mixed-cell score.

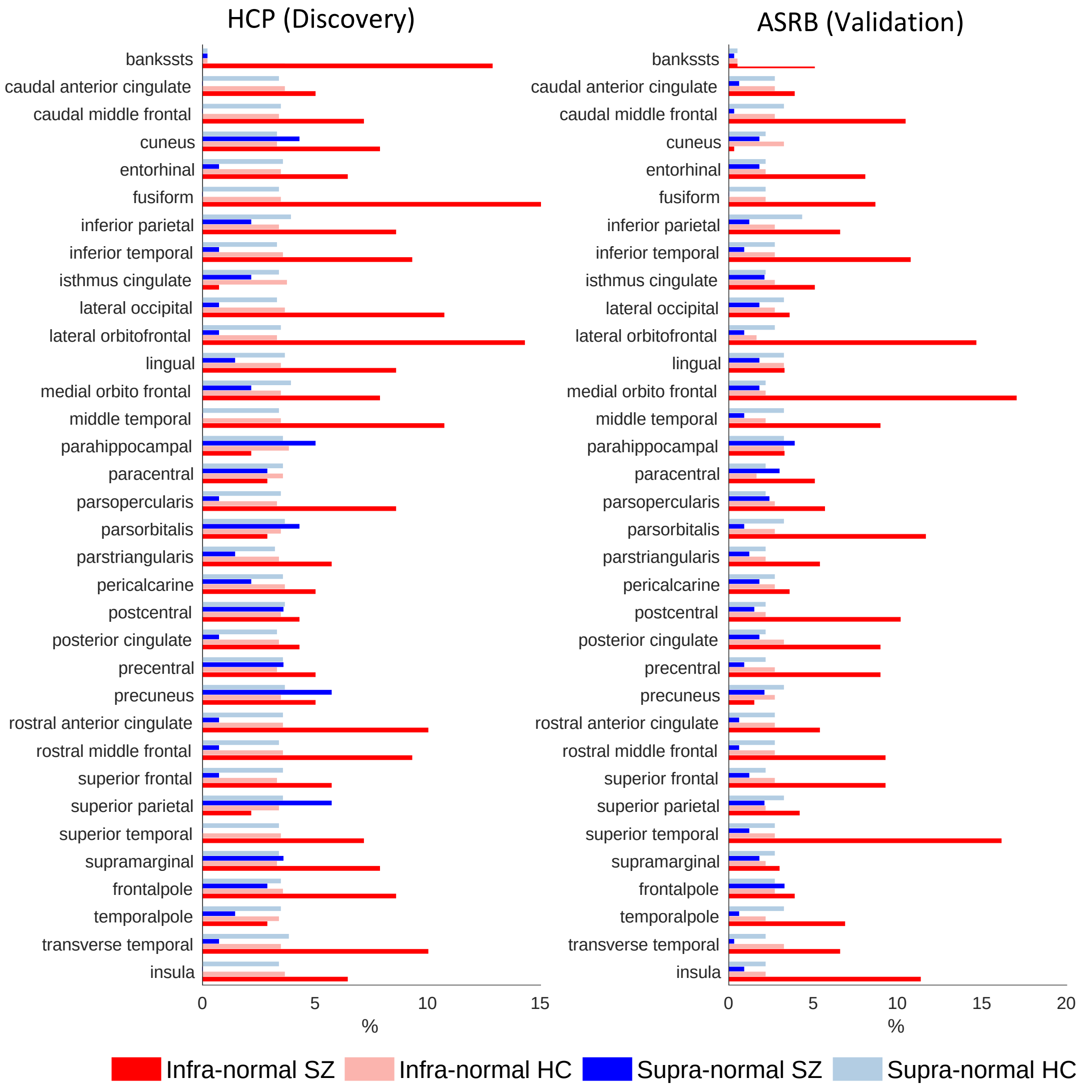

Supplementary Figure 4. Percentage of individuals residing outside the normative range in regional cortical thickness. The horizontal axis shows the percentage of individuals deemed to reside within the infra-normal (red) and supra-normal (blue) range, respectively for individuals with schizophrenia and healthy controls. The vertical axis represents cortical regions. Bar plots were generated separately for each gray matter measure, including cortical thickness, surface area and gyrification.

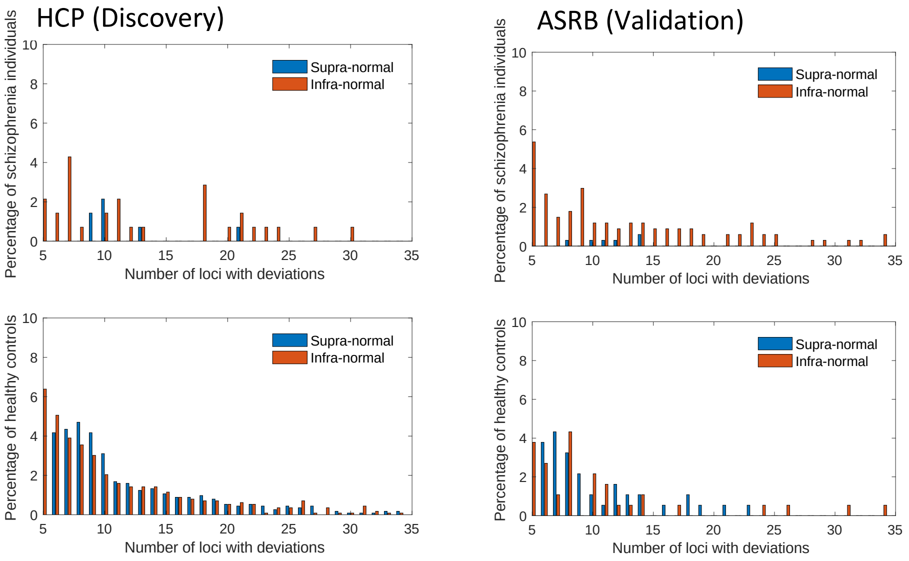

Supplementary Figure 5. Distribution of cortical loci with deviations. *Bar plots show the distribution of the total number of regions per individual with supra-normal (blue bar) and infra-normal (red) deviations from the normative model. Separate bar plots are shown for the healthy comparison individuals (first row) and individuals with schizophrenia (second row) and for each gray matter measure: cortical thickness (first column), surface area (second column) and gyrification (third column).*

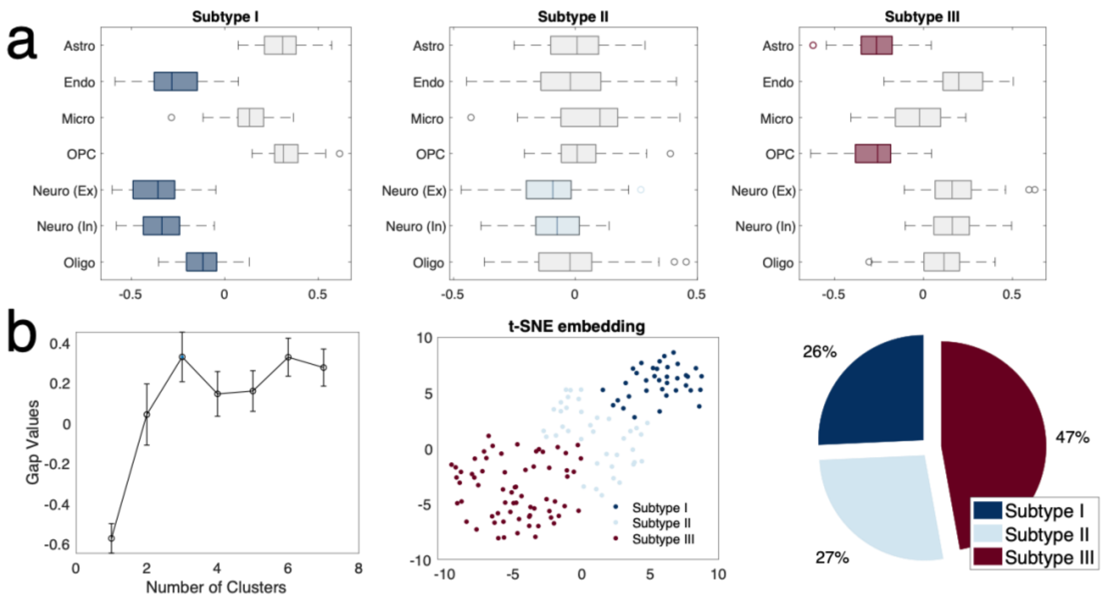

Supplementary Figure 6. Cell-based patient subtypes in the Discovery (HCP) cohort. (a) Person-specific correlations between cortical deviations with interregional cell type-specific gene expression profiles were clustered into three cell-based subtypes. Boxplots show characteristic gene expression-CTh deviation association profiles for each subtype. Colored boxes denote negative associations (i.e., where CTh loss maps onto higher cell type gene expression) that significantly differ from zero (pFDR<0.05). Box edges indicate 25th and 75th percentiles of inter-individual variation in standardized gene expression for each cell type. Central mark indicates median, whisker extend to the most extreme datapoints, and circles denote outliers. (b) The line plot demonstrates that the maximum gap criterion occurs at three clusters, which is more than one standard error from the next maximum gap value. The middle plot shows the t-Distributed Stochastic Neighbor Embedding (t-SNE), whereby person-specific points are embedded into three clusters in a way that respects similarities between points. The pie chart displays the portion of schizophrenia subjects comprising each subtype.

Illness factors and broad cell-type stratification.

To examine whether broad cellular stratifications were driven by demographic or illness-related factors, separate general linear models (GLMs) tested whether the following variables significantly differed between individuals residing in Subtype I, Subtype II and Subtype III: age, sex, illness duration, IQ, positive and negative symptom severity. Subtype descriptives for each variable by subtype are shown in turn (Supplementary Table 3). No significant group differences were observed for any demographic/illness variable examined ($p>0.05$).

Supplementary Table 6. Demographic and illness factors grouped by broad cellular subtype

**ASRB (Validation)**		**Subtype I**	**Subtype II**	**Subtype III**
**Variable**	**Covariates**	Mean (SD)	Mean (SD)	Mean (SD)
Age	Sex	41.40 (11.29)	39.38 (11.15)	39.02 (10.03)
Sex	Age	30 (41%) F	36 (25%) F	34 (29%) F
IQ	Age and sex	105.89 (13.83)	104.24 (15.26)	104.72 (16.24)
Illness duration	Sex	17.14 (10.70)	15.74 (10.36)	14.84 (8.45)
Positive symptoms	Age and sex	7.25 (3.30)	7.72 (3.87)	7.93 (3.75)
Negative symptoms	Age and sex	25.38 (18.39)	25.88 (18.87)	25.96 (19.55)

Supplementary Table 7: Summary of raw PRS for each GWAS and subset of genes.

	PGC 2020		CLOZUK 2018	
**PRS Subset**	**Mean PRS - Conservative**	**Mean PRS - Liberal**	**Mean PRS - Conservative**	**Mean PRS - Liberal**
Genome-wide	-0.006190425	-0.006190425	-0.003385653	-0.003385653
Astro	-0.005215772	-0.004834219	-0.003115134	-0.003166321
Endo	-0.005133281	-0.004308372	-0.004447465	-0.00384914
Micro	-0.005774609	-0.006410982	-0.002243353	-0.003810285
OPC	-0.006648606	-0.006584999	-0.004494582	-0.004455347
Neuro-Ex	-0.006696077	-0.006536818	-0.00275039	-0.0027937
Neuro-In	-0.0066699	-0.006572117	-0.002809306	-0.003232926
Oligo	-0.005033048	-0.005811155	-0.003182241	-0.00361246

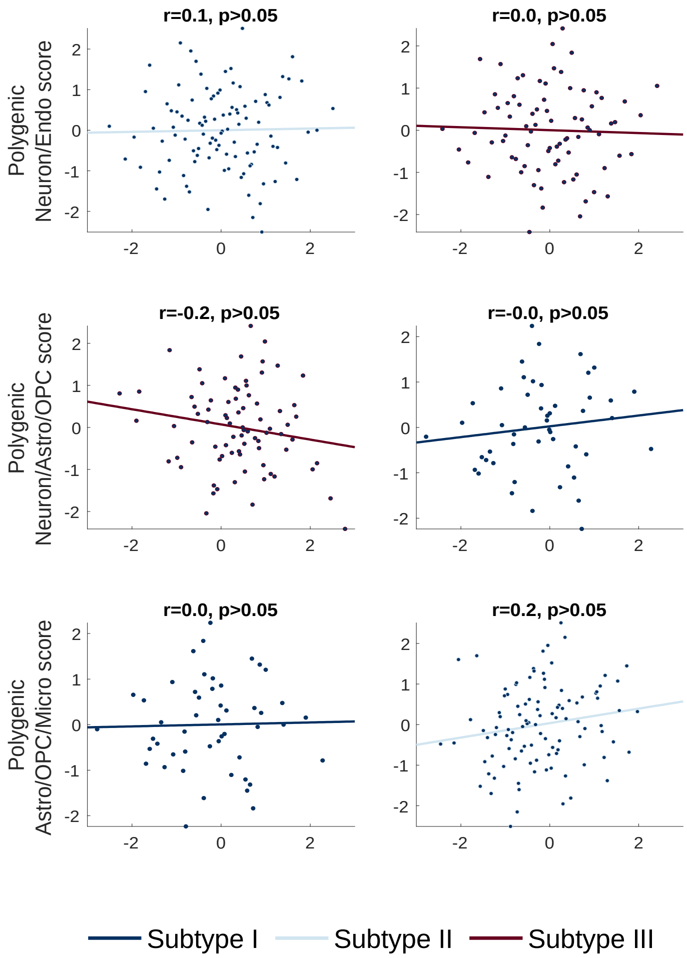

Supplementary Figure 7. Specificity analyses. Scatterplots present correlations between polygenic scores (y-axes) and cortical thickness deviations (x-axes), colored according to cell-based subtype (e.g., dark blue datapoints represent individuals comprising Subtype I). Shaded areas represent the 95% confidence interval, colored according to subtype. In specificity analyses, we demonstrate that regional cortical deviations do not significantly covary with polygenic scores in individuals comprising irrelevant subtypes. For example, scatterplots in the first row show that variability in polygenic neuronal/endothelial scores do not covary with cortical deviations in schizophrenia individuals comprising Subtype-II or Subtype III.

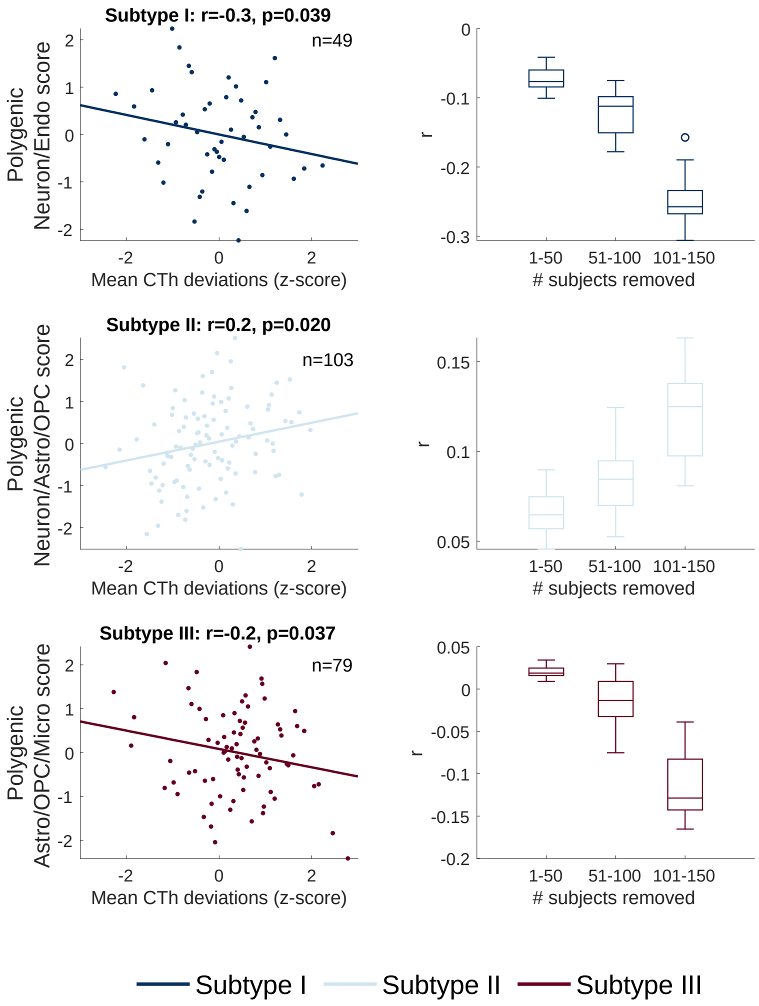

Supplementary Figure 8. Validation results using a liberal genic boundary. Scatterplots present correlations between polygenic scores (y-axes) and cortical thickness deviations (x-axes), colored according to cell-based subtype (e.g., dark blue datapoints represent individuals comprising Subtype I). Shaded areas represent the 95% confidence interval, colored according to subtype.

References

1. Van Essen, D.C., K. Ugurbil, E. Auerbach, D. Barch, et al., *The Human Connectome Project: a data acquisition perspective.* Neuroimage, 2012.
2. Lv, J., M. Di Biase, R.F.H. Cash, L. Cocchi, et al., *Individual deviations from normative models of brain structure in a large cross-sectional schizophrenia cohort.* Molecular Psychiatry, 2020.
3. Di Biase, M.A., A. Zalesky, S. Cetin-Karayumak, Y. Rathi, et al., *Large-Scale Evidence for an Association Between Peripheral Inflammation and White Matter Free Water in Schizophrenia and Healthy Individuals.* Schizophrenia Bulletin, 2020.
4. Fischl, B. and A.M. Dale, *Measuring the thickness of the human cerebral cortex from magnetic resonance images.* Proc Natl Acad Sci U S A, 2000.
5. Desikan, R.S., F. Segonne, B. Fischl, B.T. Quinn, et al., *An automated labeling system for subdividing the human cerebral cortex on MRI scans into gyral based regions of interest.* Neuroimage, 2006.
6. Fischl, B., D.H. Salat, E. Busa, M. Albert, et al., *Whole brain segmentation: automated labeling of neuroanatomical structures in the human brain.* Neuron, 2002.
7. Fortin, J.-P., N. Cullen, Y.I. Sheline, W.D. Taylor, et al., *Harmonization of cortical thickness measurements across scanners and sites.* NeuroImage, 2018.
8. Huizinga, W., D.H.J. Poot, M.W. Vernooij, G.V. Roshchupkin, et al., *A spatio-temporal reference model of the aging brain.* Neuroimage, 2018.
9. Seidlitz, J., A. Nadig, S. Liu, R.A. Bethlehem, et al., *Transcriptomic and cellular decoding of regional brain vulnerability to neurogenetic disorders.* Nature communications, 2020.
10. Romero-Garcia, R., V. Warrier, E.T. Bullmore, S. Baron-Cohen, et al., *Synaptic and transcriptionally downregulated genes are associated with cortical thickness differences in autism.* Molecular Psychiatry, 2019.
11. Seidlitz, J., F. Váša, M. Shinn, R. Romero-Garcia, et al., *Morphometric similarity networks detect microscale cortical organization and predict inter-individual cognitive variation.* Neuron, 2018.
12. Darmanis, S., *A survey of human brain transcriptome diversity at the single cell level.* Proc. Natl Acad. Sci. USA, 2015.
13. Zhang, Y., *Purification and characterization of progenitor and mature human astrocytes reveals transcriptional and functional differences with mouse.* Neuron, 2016.
14. Habib, N., *Massively parallel single-nucleus RNA-seq with DroNc-seq.* Nat. Methods, 2017.
15. Lake, B.B., *Integrative single-cell analysis of transcriptional and epigenetic states in the human adult brain.* Nat. Biotechnol., 2018.
16. Li, M., *Integrative functional genomic analysis of human brain development and neuropsychiatric risks.* Science, 2018.
17. The Schizophrenia Working Group of the Psychiatric Genomics, C., S. Ripke, J.T.R. Walters and M.C. O'Donovan, *Mapping genomic loci prioritises genes and implicates synaptic biology in schizophrenia.* medRxiv, 2020.

18

18. Pardiñas, A.F., P. Holmans, A.J. Pocklington, V. Escott-Price, et al., *Common schizophrenia alleles are enriched in mutation-intolerant genes and in regions under strong background selection.* Nature Genetics, 2018.
19. de Leeuw, C.A., J.M. Mooij, T. Heskes and D. Posthuma, *MAGMA: generalized gene-set analysis of GWAS data.* PLoS Comput Biol, 2015.
20. Reay, W.R. and M.J. Cairns, *Pairwise common variant meta-analyses of schizophrenia with other psychiatric disorders reveals shared and distinct gene and gene-set associations.* Translational Psychiatry, 2020.
21. Wray, N.R., S. Ripke, M. Mattheisen, M. Trzaskowski, et al., *Genome-wide association analyses identify 44 risk variants and refine the genetic architecture of major depression.* Nature genetics, 2018.
22. De Leeuw, C.A., B.M. Neale, T. Heskes and D. Posthuma, *The statistical properties of gene-set analysis.* Nature Reviews Genetics, 2016.
23. de Leeuw, C.A., S. Stringer, I.A. Dekkers, T. Heskes, et al., *Conditional and interaction gene-set analysis reveals novel functional pathways for blood pressure.* Nature communications, 2018.
24. Reay, W.R., J.R. Atkins, V.J. Carr, M.J. Green, et al., *Pharmacological enrichment of polygenic risk for precision medicine in complex disorders.* Scientific reports, 2020.
25. Reay, W.R., J.R. Atkins, Y. Quidé, V.J. Carr, et al., *Polygenic disruption of retinoid signalling in schizophrenia and a severe cognitive deficit subtype.* Molecular Psychiatry, 2020.
26. Choi, S.W. and P.F. O'Reilly, *PRSice-2: Polygenic Risk Score software for biobank-scale data.* Gigascience, 2019.
27. Ripke, S., J.T. Walters and M.C. O'Donovan, *Mapping genomic loci prioritises genes and implicates synaptic biology in schizophrenia.* medRxiv, 2020.
28. Pardiñas, A.F., P. Holmans, A.J. Pocklington, V. Escott-Price, et al., *Common schizophrenia alleles are enriched in mutation-intolerant genes and in regions under strong background selection.* Nature genetics, 2018.
